# Hypothalamic‐related neural mechanism in lifelong premature ejaculation and trends following selective serotonin reuptake inhibitor administration

**DOI:** 10.1111/cns.13882

**Published:** 2022-06-10

**Authors:** Bowen Geng, Ming Gao, Ke Xu, Shuming Zhang, Peng Liu

**Affiliations:** ^1^ Life Science Research Center, School of Life Science and Technology Xidian University Shaanxi China; ^2^ Engineering Research Center of Molecular and Neuro Imaging Ministry of Education, School of Life Science and Technology Xidian University Xi'an Shaanxi China; ^3^ Department of Urology Xi'An Daxing Hospital Affiliated to Yan'an University China

## Abstract

Lifelong premature ejaculation patients had altered structural and functional parameters of the hypothalamus compared with healthy controls. The patients showed increased hypothalamus‐related functional connectivity in several regions one hour after dapoxetine administration. The changing trends of the brain functional connectivity after dapoxetine administration possibly provided important information about the selective serotonin reuptake inhibitor effects on the functional neural system.

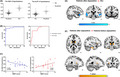

Dear Editor,

As one of the most prevalent male sexual dysfunctions worldwide, lifelong premature ejaculation (PE) is characterized by ejaculation that always occurs before or within 1 min of vaginal penetration from the first sexual experience and inability to delay ejaculation on all vaginal penetrations.^1^ Although its pathophysiological mechanisms have not yet been entirely elucidated, a number of treatment options for PE are available currently. In addition to behavioral techniques and psychotherapy, there are other widely used therapeutic options such as local anesthetics, phosphodiesterase type 5 inhibitors, and selective serotonin reuptake inhibitor (SSRI).^2^ Dapoxetine is the first oral SSRI approved for the treatment of PE in many countries. It is characterized by rapid absorption and short initial half‐life, with maximal plasma concentrations reaching a peak about 1 hour after oral administration.^3^ Several clinical trials reported that oral dapoxetine 30 or 60 mg induced a significant improvement in intravaginal ejaculation latency time (IELT) when compared with placebo.^4^

The abnormal neural mechanisms, especially for the altered structural and functional pattern in lifelong PE patients, were widely discussed based on the development of magnetic resonance imaging (MRI) technology. As a multifunctional brain region, the hypothalamus is considered to control important body functions, such as thermoregulation, sleep, and even sexual behavior.^5^ Prenatal SSRI exposure affects the developing hypothalamic–pituitary–adrenal system in infants.^6^ Interestingly, the hypothalamus‐seeded functional connectivity (FC) alterations between the lifelong PE patients were discovered in the recent study.^7^ Nevertheless, few studies explored the neural mechanism changes of PE patients after SSRI administration.

We aim to explore whether the hypothalamus‐related structural and functional parameters are altered in patients compared to healthy controls (HCs), and what are the changing trends after dapoxetine administration.

The study was in line with the Declaration of Helsinki, approved by the local hospital's ethics committee, and registered on ClinicalTrials.gov. We enrolled 25 right‐hand‐dominant lifelong PE patients and 31 matched HCs. Exclusion and inclusion criteria were in line with the International Society for Sexual Medicine's guidelines and previous works.^1^, ^8^ The demographic and clinical features were obtained from all subjects including Premature Ejaculation Diagnostic Tool (PEDT) score, IELT, International Index of Erectile Function (IIEF)‐5 score, Self‐Rating Anxiety Scale (SAS), and Self‐Rating Depression Scale (SDS) (Table 1). The normality of clinical variables distribution was assessed by D'Agostino and Pearson omnibus normality test. The variables were compared across groups using independent sample t‐test or, if the variables do not exhibit a Gaussian distribution, Mann–Whitney t‐test. The resting‐state functional and structural MRI scans were performed on all subjects. Then, functional scans of PE patients were collected again 1 hour after dapoxetine administration. All MRI scans were acquired with a 3.0 T MRI system (Excite, General Electric, Milwaukee, WI) at a local hospital.

**Table 1:** Demographic and clinical characteristics of lifelong PE patients and HCs

	PE (*n* = 25)	HCs (*n* = 31)	*p*‐value
Ages (years)	30.76 ± 0.73	31.71 ± 0.51	0.2796
PEDT score	16.20 ± 0.45	0.77 ± 0.26	0.000***
IELT (s)	48.20 ± 2.12	672.60 ± 60.09	0.000***
SAS	44.48 ± 0.72	30.48 ± 0.30	0.000***
SDS	43.64 ± 1.02	30.52 ± 0.38	0.000***

*Note*: Data were expressed as the mean ± standard error of mean (SEM) (range values).

Abbreviations: HCs, healthy controls; IELT, intravaginal ejaculatory latency time; IIEF‐5, International Index of Erectile Function; PE, premature ejaculation.

***p < 0.001 by independent sample t‐test.

The processing of structural and functional data was performed by Statistical Parametric Mapping 12, Computational Anatomy Toolbox 12, and DPABI toolbox. After extracting the mean gray matter volume and mean amplitude of low‐frequency fluctuations (ALFF) value of the hypothalamus, the statistical differences between the patients and HCs were calculated by unpaired t‐test. The statistical threshold was set at *p* < 0.05. Then, accuracy and area under curve (AUC) were applied to evaluate the classification performance of these structural and functional parameters. Moreover, the correlation analysis was then examined between these parameters and clinical features (e.g., PEDT and IELT) at *p* < 0.05. For the FC analysis, a two‐sample t‐test was applied to examine the group differences between the PE patients before dapoxetine and HCs, as well as the PE patients after dapoxetine and HCs. Moreover, paired t‐test was used to calculate the differences between PE patients before and after dapoxetine. The significant level was set at *p* < 0.05 (false discovery rate [FDR] corrected).

Our results found that PE patients had decreased gray matter volume (*p* < 0.0001) and increased mean ALFF value (*p* < 0.0001) in the hypothalamus compared with HCs (Figure 1A). The area under the ROC curve was 0.93 for gray matter volume and 0.85 for ALFF value (Figure 1B), suggesting that these parameters might accurately distinguish PE patients from HCs. In addition, the PEDT score of PE patients negatively correlated with the gray matter volume of the hypothalamus (r = −0.51, *p* = 0.009) and positively correlated with the mean ALFF value in the hypothalamus (r = 0.48, *p* = 0.01; Figure 1C).

**FIGURE 1:**
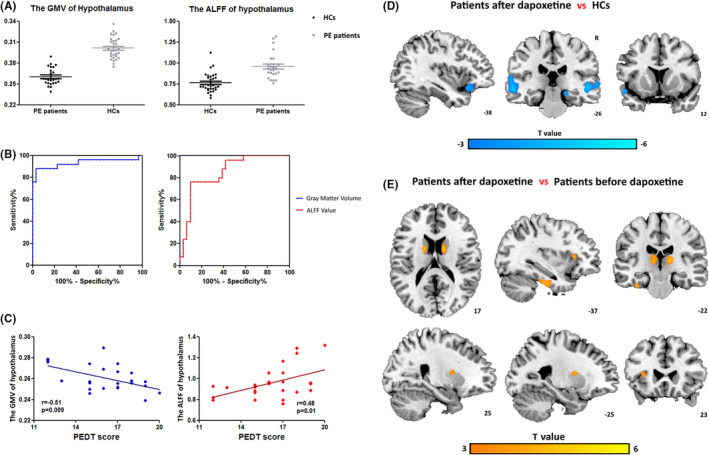
(A) Comparison of GMV and ALFF value in hypothalamus between lifelong PE patients and HCs. (B) ROC curve of the model. The red line is the ROC curve of the ALFF value, and the blue line is the ROC curve of GMV. (C) Correlations between the hypothalamus‐related imaging parameters and PEDT score in lifelong PE patients. (D) Brain regions showing significantly altered hypothalamus‐related FC between the lifelong PE patients after dapoxetine administration and HCs. (E) Lifelong PE patients after dapoxetine administration showed significantly altered hypothalamus‐related FC in certain brain regions compared to patients before dapoxetine administration. Abbreviations: GMV, gray matter volume; ALFF, amplitude of low‐frequency fluctuations; ROC, receiver operating characteristic; PEDT, Premature Ejaculation Diagnostic Tool; R, right hemisphere

The differences in the hypothalamus‐seeded FC between the PE patients and HCs were similar to the previous study.^7^ PE patients after dapoxetine administration showed decreased hypothalamus‐related FC in the bilateral superior/middle temporal gyrus, left orbital frontal cortex, left superior temporal pole, and right parahippocampal compared to HCs (Figure 1D). Additionally, PE patients after dapoxetine administration showed increased hypothalamus‐related FC in the bilateral caudate, bilateral putamen, bilateral thalamus, left fusiform, and left insula compared to patients before dapoxetine administration (Figure 1E).

Serotonin (5‐HT) plays an integral part in neurodevelopment. What is particularly noteworthy is that the expression of 5‐HT receptors is primarily detected in the caudate, putamen, and thalamus,^9^ and it may be one of the reasons why PE patients showed significant amelioration of anomalous hypothalamus‐related FC in such regions after dapoxetine administration.

Our study is not without limitations. First, although the current findings were able to reproduce in a separate independent sample, further replication is warranted. Second, we could not acquire the structural MRI data and symptomatic improvement of patients 1 hour after dapoxetine administration. Finally, several social factors (living environment, marriage status, and incidental alcohol consumption) and duration of treatment may influence outcomes, so our findings should be considered preliminary and require further validation, and future studies should explore whether current findings are specific to long‐term treatment underlying the longitudinal imaging data.

To our knowledge, this is the first MRI study to report the alteration of hypothalamus‐related FC in lifelong PE patients after SSRI administration. We revealed that PE patients had abnormal structural and functional parameters of the hypothalamus, which might be considered the neurobiological underpinning of PE. Furthermore, the changing trends of the brain functional connectivity after dapoxetine administration possibly provided important information about the SSRI effects on the functional neural system and facilitated the development of long‐term SSRI treatment.

## CONFLICT OF INTEREST

The authors have no conflicts of interest to declare.

## Data Availability

The data that support the findings of this study are available on request from the corresponding author. The data are not publicly available due to privacy or ethical restrictions.

## References

[1] Althof SE , McMahon CG , Waldinger MD , et al. An update of the International Society of Sexual Medicine's guidelines for the diagnosis and treatment of premature ejaculation (PE). Sexual Medicine Jun 2014;2(2):60–90. 25356302 10.1002/sm2.28 PMC4184677

[2] Saitz TR , Serefoglu EC . Advances in understanding and treating premature ejaculation. Nat Rev Urol. 2015;12(11):629‐640. 26502991 10.1038/nrurol.2015.252

[3] Modi NB , Dresser MJ , Simon M , Lin D , Desai D , Gupta S . Single‐ and multiple‐dose pharmacokinetics of dapoxetine hydrochloride, a novel agent for the treatment of premature ejaculation. J Clin Pharmacol. 2006;46(3):301‐309. 16490806 10.1177/0091270005284850

[4] McMahon CG , Althof SE , Kaufman JM , et al. Efficacy and safety of Dapoxetine for the treatment of premature ejaculation: integrated analysis of results from five phase 3 trials. J Sex Med. 2011;8(2):524‐539. 21059176 10.1111/j.1743-6109.2010.02097.x

[5] Saper CB , Lowell BB . The hypothalamus. Curr Biol. 2014;24(23):R1111‐R1116. 25465326 10.1016/j.cub.2014.10.023

[6] Pawluski JL , Brain UM , Underhill CM , Hammond GL , Oberlander TF . Prenatal SSRI exposure alters neonatal corticosteroid binding globulin, infant cortisol levels, and emerging HPA function. Psychoneuroendocrinology. 2012;37(7):1019‐1028. 22177580 10.1016/j.psyneuen.2011.11.011

[7] Gao M , Feng NN , Wu JY , et al. Altered functional connectivity of hypothalamus in lifelong premature ejaculation patients. J Magn Reson Imaging. 2020;52(3):778‐784. 32068927 10.1002/jmri.27099

[8] Geng BW , Gao M , Wu JY , et al. Smaller volume and altered functional connectivity of the amygdala in patients with lifelong premature ejaculation. Eur Radiol. 2021;31(11):8429‐8437. 33928418 10.1007/s00330-021-08002-9

[9] Wirth A , Holst K , Ponimaskin E . How serotonin receptors regulate morphogenic signalling in neurons. Prog Neurobiol. 2017;151:35‐56. 27013076 10.1016/j.pneurobio.2016.03.007

